# Mr. Henry on the Digitalis, and Frictions with Opium

**Published:** 1799-09

**Authors:** Thomas Henry

**Affiliations:** Manchester


### *To the Editors of the Medical and Phyfical Journal.*

GENTLEMEN,

ONE great advantage of your valuable publication is, that it admits of the communication of practical facts, the knowledge of which may be ufeful to medical men, without the neceffity of entering into long details, for which few practitioners have time or inclination.

The ufe of digitalis purpurea, in pulmonary difeafes, has, for fome years paft, been no novelty in this town; and the gentlemen who have employed it, have commonly joined it with opium—a combination by which the inconveniences of the two medicines, often experienced when they are given feparately, are happily corrected. Perhaps the Materia Medica contains no remedy which affords more relief to, and which even cures, harraffing coughs, attended with inflammatory fymptoms, that often induce phthifis, than the digitalis; though I cannot fay I have ever known phthifis, when really formed, to be removed by it..—From its remarkable effects in diminifhing the force of the arterial fyftem, Dr. FERRIAR was led to adminifter it, with great fuccefs, in cafes of active hæmorrhage, and, by parity of reafoning, I laft winter employed it with advantage, in a cafe of acute rheumatifm.

A gentleman's fervant, of thin habit, and narrow cheft, by carelefly expofing himfelf to the cold of laft winter, was repeatedly attacked by a bad cough, which yielded to the common remedies.—On the firft of December, by inattention to thofe precautions of warm cloathing, &c. which had been recommended to him, his cough returned, attended with acute pain in his breaft, affecting his refpiration, fo as to threaten peripneumony;—blood, which proved fizy, was taken from his arm; antimonials, demulcents with nitre, and aperients were exhibited; the pain now quitted his breaft, and attacked his fhoulder, from whence, during the ensuing enſuing days, it removed alternately into the higher and lower limbs, attended with ſuch ſwelling of the hands and feet, as clearly pointed out the caſe to be rheumatic. In addition to the above-mentioned medicines, he, on the evening of the fourth, took pulv. Ipecac. c. ſcrup. j. which was repeated every night till the eighth, when, the pain increaſing in violence, the antimonials were omitted, and ten grains of the ſame powder were given every ſix hours. The remedies that had been uſed, cauſed profuſe perſpiration, but without producing the leaſt abatement of his pain, and his pulſe beat 140 ſtrokes in the minute. My experience had taught me not to expect much relief from repeated bleeding in this diſeaſe, yet it ſeemed neceſſary to reduce the action of the veſſels. From the well-known efficacy of digitalis in diminiſhing the frequency of the pulſe, it occurred to me, to make trial of it, on this occaſion; and the cough being very troubleſome, and pain acute, I determined to join opium with it; accordingly, at three o'clock in the afternoon, December 9th, he took a pill compoſed of opii gr. j, pulv. fol. digital. purpur. gr. ſs. On viſiting him in the evening, his pulſe was found conſiderably leſs frequent, and he thought his pain and cough not quite ſo ſevere. He was directed to repeat the pill every eight hours. The next morning all his ſymptoms were ameliorated, and his pulſe reduced to about 90. The ſame plan was therefore continued, with evident advantage, attention being paid to avoid conſtipation, till the fourteenth, when the rheumatiſm being removed, and his pulſe reduced nearly to the natural ſtandard, the digitalis was omitted, and he was ordered a decoction of bark with antimonial wine; but his cough being at times troubleſome, he took a pill of opium and camphor twice a day, for two or three days longer. On the 18th, the cough being ſtill teazing, a bliſter was applied between the ſhoulders; and, by it, and a continuance of the bark, for a few more days, he was completely cured.

It is with pleaſure I am able to bear teſtimony to the good effects of opiate friction, as recommended by my worthy friend, Mr. WARD, in the fifth Number of your Journal.

A young lady of about thirteen years of age, much harraſſed with aſcarides, had been removed, on the death of a brother by typhus, from this town to Warrington. In a few days, ſymptoms of typhus attacked her; but, inſtead of proper meaſures being taken, ſhe was encouraged to walk out; and growing worſe, her friend, with whom ſhe had been placed, brought her, on the tenth day of the fever, twenty miles in a ſtage-coach, to her parents. On the evening of this day, July 28th, I viſited her, and found her in a ſtate of great irritability, with a rapid pulſe, conſiderable heat, and fallen countenance; her bowels being conſtipated, ſhe was, with great great difficulty, prevailed on to take a powder confifting of calomel and James's powder, of each one grain, rhubarb and opiate powder, of each four grains: this procured three loofe ftools, in which a great number of afcarides were difcharged; fhe now, with a degree of obftinacy I have feldom witneffed, protefted againft taking any medicine, or even any appropriate food. On the thirtieth, a diarrhœa prevailing to an alarming degree, ten drops of laudanum, concealed in a cup of coffee, were given without her knowledge; and this fingle dofe was the only medicine which, during three days, we were able to adminifter. She was finking faft, her pulfe very rapid and feeble, and fhe had a tendency to delirium. Wine, which in this ftate, feemed highly requifite, fhe pofitively rejected; and I defpaired of being able to fucceed by internal means. I therefore refolved to try the external application of opium. The following ointment was directed to be divided into two parts, and one of them to be rubbed into the thigh of the patient, by an affiftant, whofe hand was ordered to be covered with a foft bladder:

℞ Opii fubtiliffime pulv. fcrup. i.
Adipis fuill. drachm iij.
Camphor. fcrup. fs. M. and divide in chartalus duas.

This application feemed to have fome fedative effect, aad was therefore repeated in the evening. The next morning, Auguft 1ft, I had the fatiffaction to find that fhe had had fome comfortable fleep; but the loofenefs recurring, we contrived to convey ten drops of laudanum again in coffee. The ointment, with an increafe of ten grains of opium was continued, and as fhe appeared more compofed, I determined to abide fteadily by it. The good effects were each time more evident; fleep almoft conftantly followed the application of the ointment, though no more laudanum was given internally; her pulfe and the intenfity of heat were gradually reduced, the diarrhœa was ftopped, and on the fourth day from the commencement of this treatment fhe began to take fome wine, acidulated with fulphuric acid. The friction on different parts was perfifted in till the 8th of Auguft, inclufively; when the urine having, for the two laft days, depofited a proper fediment, and every fymptom of fever having difappeared, it was difcontinued.

With much refpect, I am,

Gentlemen,

Your obedient fervant,

THOMAS HENRY.

MANCHESTER,

